# *TOX3* Promotes Ovarian Estrogen Synthesis: An RNA-Sequencing and Network Study

**DOI:** 10.3389/fendo.2020.615846

**Published:** 2021-02-24

**Authors:** Yuanyuan Man, Rusong Zhao, Xueying Gao, Yue Liu, Shigang Zhao, Gang Lu, Wai-Yee Chan, Peter C. K. Leung, Yuehong Bian

**Affiliations:** ^1^ Center for Reproductive Medicine, Cheeloo College of Medicine, Shandong University, Jinan, China; ^2^ Key Laboratory of Reproductive Endocrinology of Ministry of Education, Shandong University, Jinan, China; ^3^ Shandong Key Laboratory of Reproductive Medicine, Jinan, China; ^4^ Shandong Provincial Clinical Research Center for Reproductive Health, Jinan, China; ^5^ National Research Center for Assisted Reproductive Technology and Reproductive Genetics, Shandong University, Jinan, China; ^6^ CUHK-SDU Joint Laboratory on Reproductive Genetics, School of Biomedical Sciences, The Chinese University of Hong Kong, Hong Kong, China; ^7^ Department of Obstetrics and Gynaecology, BC Children’s Hospital Research Institute, University of British Columbia, Vancouver, BC, Canada

**Keywords:** TOX3/TNRC9, breast cancer, KGN, estrogen, RNA-Seq, GSEA

## Abstract

**Background:**

Women who undergo chronic exposure to excessive estrogen are at a high risk of developing breast cancer. *TOX3* has been reported to be highly expressed in breast tumors and is closely related to estrogen receptors. However, the effect of TOX3 on estrogen synthesis remains poorly understood.

**Methods:**

Using lentiviruses as a vector, we stably overexpressed TOX3 in the ovarian granulosa cell line KGN, the cells where estradiol is primarily produced, to investigate its role in estrogen production as well as cell viability and apoptosis. RNA-Sequencing was applied to uncover the global gene expression upon TOX3 overexpression.

**Results:**

We observed an increased level of cell viability and a reduced cell apoptosis rate after TOX3 overexpression, and the level of estradiol in the cell culture supernatant also increased significantly. Gene set enrichment analysis of the transcriptome showed that the ovarian steroidogenesis pathway was significantly enriched. Similarly, pathway mapping using the Kyoto Encyclopedia of Genes and Genomes and Gene Ontology analyses also showed that TOX3 overexpression affects the ovarian steroidogenesis pathway. Further experiments showed that upregulated *FSHR*, *CYP19A1*, and *BMP6* accounted for the enhanced estrogen synthesis.

**Conclusion:**

Our study demonstrated that TOX3 quantitatively and qualitatively stimulates estrogen synthesis by enhancing estrogen signaling pathway–related gene expression in ovarian granulosa cells. These findings suggest that TOX3 may play a vital role in the pathogenesis of breast cancer.

## Introduction

Breast cancer is one of the most commonly reported malignant tumors and a leading cause of death in women (1, 2). The pathogenesis and development of breast cancer are associated with high levels of estrogen; it has been reported that 84% of breast cancers express estrogen and progesterone receptors, thus indicating the crucial role of the hormone in the development of breast cancer (3). Generally, a high level of circulating estrogen is considered a major risk factor in premenopausal women (4, 5). For postmenopausal women, an elevated incidence of breast cancer was observed in women receiving hormonal therapy (4, 5). Besides, an excess body weight of women also increases the risk of developing breast cancer after menopause due to adipose tissue-related higher activity of aromatase (6, 7). Specifically, an increased level of endogenous estrogens was associated with a higher risk for breast cancer in women postmenopausal (6, 8). For women throughout the reproductive years, estrogen exposure is likely to be associated with higher risk of breast cancer. Estrogen binds to estrogen receptors (ER) to activate downstream signal transduction pathways, thereby modulating cell proliferation, differentiation, and apoptosis (9). Abnormalities in the estrogen signaling pathway disturb gene expression and induce abnormal proliferation, migration, and invasion of breast cancer cells, thus promoting the malignant evolution of breast cancer.

*TOX3*, or TOX high-mobility group (HMG)-box protein group family member 3, has been previously identified as a breast cancer susceptibility gene in a large-scale genome-wide association study (10). TOX3 can interact with cAMP response element–binding protein (CREB) to regulate Ca^2+^-dependent transcription and with CITED1 to promote the transcription of estrogen response elements in the neuron system (11, 12). In the mammary glands of mice, TOX3 was found to be highly expressed during breast cancer, and its expression was correlated with clinical tumor, nodes, and metastases stage progression (13, 14). Overexpression of TOX3 promotes invasion and metastasis in breast cancer cells and enhances their invasive ability by downregulating BRCA1 expression, whereas tumorigenesis and tumor formation are significantly reduced after TOX3 downregulation (13). This strongly suggests that TOX3 plays an important role in the development of breast cancer; however, whether TOX3 promotes the development of breast cancer through its involvement in the regulation of estrogen synthesis is still unclear.

Estrogen is synthesized mainly by granulosa cells in the ovaries (15) and plays an important role in various physiological processes including sexual development, regulation of energy metabolism, and stress responses (16). In the present study, we investigated the biological function of TOX3 in estrogen production in ovaries in order to further explain the relationship between TOX3 and breast cancer, using KGN cells, the most commonly used human granulosa cell line, which are capable of estrogen synthesis (17).

## Materials and Methods

### Cell Culture

The human granulosa-like tumor cell line, KGN, was donated from the RIKEN BioResource Research Center in Japan (17). The normal granulosa cell line SVOG was a gift from Prof. Peter C.K. Leung of University of British Columbia in Canada to The Chinese University of Hong Kong-Shandong University (CUHK-SDU) Joint Laboratory on Reproductive Genetics. Cells were cultured in Dulbecco’s modified Eagle medium: Nutrient Mixture F-12 (DMEM/F12) (Hyclone Corporation, South Logan, UT, USA) supplemented with 10% fetal bovine serum (Hyclone Corporation) and 100 U/ml penicillin (Hyclone Corporation) and 100 mg/ml streptomycin (Hyclone Corporation) at 37°C in a humidified atmosphere containing 5% CO_2_.

### Lentiviral Infection

Control (lentiviral-control, LV-CON) and TOX3 (lentiviral-TOX3, LV-TOX3) overexpression lentiviruses (GeneChem Company, Shanghai, CHN) or Cas9 and sgRNA of control/TOX3 (LV-CON/LV-KO) carrying the puromycin resistance gene were synthesized. The sequence of sgRNA for TOX3 knock out was 5’-TGCGTTCTTCGCTGCCAGTG-3’. Lentiviruses were added at a ratio of 1:200 into the cell culture medium when cell fusion reached 60–70%. Positive cells were screened using 2 mg/ml of puromycin for two passages.

### RNA Isolation and Quantitative Real Time-PCR

Total RNA was extracted by removing the culture medium and lysing the remaining cells using TRIzol™ Reagent (Takara Bio, Beijing, CHN) according to the manufacturer’s instructions. RNA concentration and integrity number were determined using the Agilent 2100 Bioanalyzer (Agilent Technologies, Santa Clara, CA, USA). The extracted total RNA was reverse transcribed using the PrimeScript™ RT Reagent Kit with gDNA Eraser (Takara Bio), followed by quantitative real time-PCR (qRT-PCR) using SYBR^®^ *Premix Ex Taq*™ (Takara Bio) on a LightCycler 480 System (Roche, Basel, CHE). The primers used for qRT-PCR are listed in **Table S1**. The housekeeping gene *ACTB* was used for data normalization, and the relative expression of mRNA was calculated using the 2^−ΔΔ^ *Ct* method.

### Western Blot Analysis

Cultured cells were rinsed twice with ice-cold phosphate buffer saline (PBS) and then lysed in sodium dodecyl sulfate (SDS) buffer (Beyotime Biotechnology, Shanghai, CHN) containing phenylmethanesulfonyl fluoride (PMSF) (Beyotime Biotechnology) for 5 min and collected according to the manufacturer’s instructions. The protein concentrations of the cell lysates were measured using a Pierce™ Bicinchoninic Acid Protein Assay Kit (Thermo Fisher, Waltham, MA, USA). Approximately 30 μg of protein in each lane was electrophoresed on a 12% sodium dodecyl sulfate–polyacrylamide gel electrophoresis gel and subsequently transferred to a polyvinylidene difluoride membrane (Millipore, Billerica, MA, USA). The membrane was blocked using 5% non-fat milk for 1 h at 25°C and then incubated with specific primary antibodies overnight at 4°C. The primary and secondary antibodies used in this study are listed in **Table S2**. An Immobilon ECL Ultra Western HRP Substrate (Millipore) was used to detect immunoreactive protein bands, which were visualized using the ChemiDoc MP Imaging System (Bio-Rad Laboratories, Hercules, CA, USA). ACTB was used as an internal control.

### Apoptosis Analysis Using Flow Cytometry

The apoptosis rate was analyzed using the Annexin V-PE Apoptosis Detection Kit I (BD Biosciences, San Jose, CA, USA) according to the manufacturer’s instructions. Cells stably expressing TOX3/KO or control vectors were collected accordingly by cell trypsinization and washing twice with ice-cold PBS, followed by resuspension in 100 μl 1X binding buffer and staining with 5 μl Annexin V-PE and 5 μl 7-AAD at 25°C for 15 min away from light. Then, 400 μl of 1X binding buffer was added and the apoptosis rate was analyzed using flow cytometry.

### Terminal Deoxynucleotidyl Transferase dUTP Nick End Labeling

Terminal Deoxynucleotidyl Transferase dUTP Nick End Labeling (TUNEL) staining was conducted using the One-step TUNEL Apoptosis *in situ* Assay Kit (KeyGen BioTECH, Nanjing, CHN) according to the manufacturer’s instructions. Cells with multiple DNA breaks were labeled with tetramethylrhodamine, and the nuclei were stained with DAPI (4′,6-diamidino-2-phenylindole) (Beyotime Biotechnology). Cell apoptosis was analyzed using New Dual-CCD DP80 Microscope Digital Camera (Olympus, Tokyo, Japan).

### Cell Viability Assay

Cells transfected with LV-CON or LV-TOX3/LV-KO were seeded in 96-well plates at a concentration of 20,000 cells per millimeter of culture medium. The Cell Counting Kit-8 (CCK-8) Assay (Beyotime Biotechnology) was used according to the manufacturer’s protocol to determine cell viability at time intervals 24, 48, and 72 h after cell seeding and absorbance was measured at 450 nm.

### Hormonal Test

Cultured cells were cultured in 12-well plates at a density of 1×10^5^ cells/ml in 1 ml culture medium as mentioned above. The culture medium was replaced with DMEM/F12 plus 10 nM of testosterone after 24 h when the cells reached 60–70% of cell confluency. The medium was then collected and stored at −80°C for hormonal analysis, and the remaining cells were lysed using SDS. The concentration of estradiol in the cell supernatants was measured using Estradiol III (Roche) according to the manufacturer’s instructions. The concentrations were then normalized according to the protein concentrations of the cell lysates measured using the bicinchoninic acid assay (Thermo Fisher).

### RNA Sequencing

RNA was isolated using TRIzol Reagent (Takara Bio), and the concentration and RNA integrity number were determined using an Agilent 2100 Bioanalyzer (Agilent Technologies). RNA was enriched using Oligo (dT) magnetic beads (Invitrogen, Carlsbad, CA, USA) followed by fragment screening, library building, and quantification using a Qubit 3.0 Fluorometer (Invitrogen). The samples were sequenced on Illumina using the PE150 strategy (GSE161341).

### Differentially Expressed Gene Identification and Gene Set Enrichment Analysis

The sequencing data were filtered with SOAPnuke (18) by removing 1) reads containing sequencing adapters; 2) reads whose low-quality base ratio, i.e., a base quality less than or equal to 5, was above 20%; and 3) reads whose unknown base (“N” base) ratio was more than 5%, after which clean reads were obtained and stored in the FASTQ format. The clean reads were aligned to the RefSeq hg38 genome using HISAT2 (19). Burrows-Wheeler transform was applied to align the clean reads to the reference coding gene set (20) and then the expression levels were calculated using RNA-Seq by expectation maximization (21). Further, differential expression analysis was performed using the Limma_voom (22) at *a P-value *≤ 0.05. The Empirical Bayes method (ComBat) (23) was applied to remove batch effects. GSEA was performed using the online software WEB-based Gene SeT AnaLysis Toolkit (http://www.webgestalt.org/). The corresponding Kyoto Encyclopedia of Genes and Genomes (KEGG) pathway was chosen in the functional database, and all the detected genes were uploaded according to the instructions on the website. In addition, genes with *P*-value < 0.3 and |log2FC| > 0.6 were submitted to the online resource Metascape (https://metascape.org/gp/index.html#/main/step1) to obtain gene annotation and enrichment results. Heatmaps were generated *via* the online resources HIPLOT (https://hiplot.com.cn/basic/heatmap) and GraphPad Prism 8 (GraphPad Software Inc., San Diego, CA, USA). A volcano plot was constructed with R (3.6.2).

### Statistical Analysis

Statistical analysis was performed using the Statistical Package for Social Science, version 23.0 (IBM Corp., Armonk, NY, USA). Statistical differences were analyzed using the two-tailed Student’s *t-test*. Differences were considered statistically significant at *P*<0.05. All experiments were repeated at least three times.

## Results

### Overexpression of TOX3 in KGN Cells

**Figure 1:**
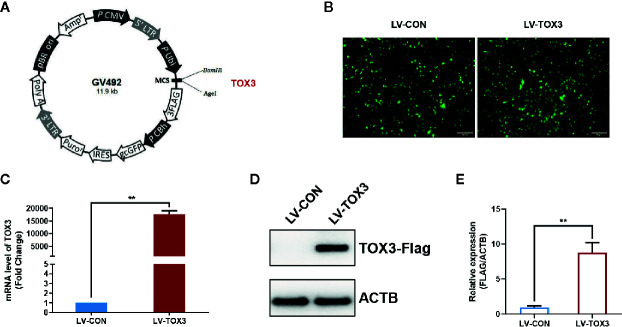
TOX3 successfully overexpressed in KGN cells. **(A)** A schematic of lentivirus plasmid. Full length of TOX3 was inserted between locus *BamHI* and *AgeI*. **(B)** Fluorescence imaging confirmed the successful infection of lentiviruses. Scale bar: 200 μm **(C)** The efficiency of overexpression was validated using qRT-PCR. The *Ct* values were normalized to the ACTB expression levels. **(D)** The efficiency of overexpression was validated *via* western blot. **(E)** Protein quantification was analyzed using ImageJ software. Data are presented as the median ± SEM and shown represent three independent experiments. **P < 0.01. Student’s *t-test*.

As a granulosa cell line with aromatase activity (17, 24), KGN cells were used to investigate estrogen synthesis after TOX3 overexpression. As shown in **Figure 1A**, the full length *TOX3* was inserted into the plasmid GV492. After screening with 2 mg/ml of puromycin for two passages, the preliminary infection efficiency was determined using green fluorescent protein (GFP) under a fluorescence microscope (**Figure 1B**). KGN cells were then lysed and subsequently subjected to qRT-PCR and western blotting. TOX3 was significantly upregulated at both the mRNA and protein levels (**Figures 1C–E**).

### TOX3 Affects Cell Viability and Promotes Estradiol Synthesis

**Figure 2:**
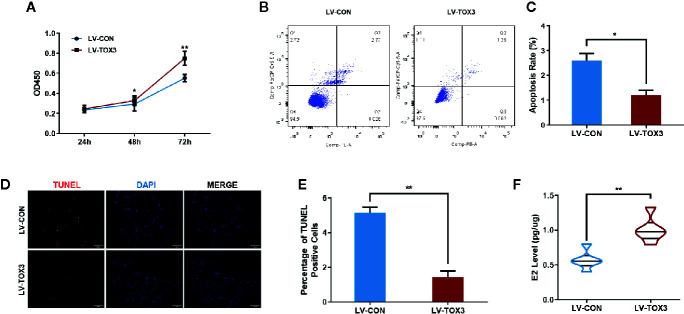
Overexpression of TOX3 increases cell viability and inhibits cell apoptosis. **(A)** The CCK-8 assay performed on KGN cells revealed that overexpression of TOX3 increased cell viability. **(B)** Apoptosis rate measured using flow cytometry. **(C)** The normalized apoptosis rate shows that KGN with overexpressed TOX3 inhibits cell apoptosis. **(D)** A TUNEL assay revealed that KGN with overexpressed TOX3 displayed less TUNEL-positive cells. Scale bar: 200 μm **(E)** Quantification of TUNEL-positive cells. **(F)** The level of estradiol was higher when TOX3 was overexpressed after being normalized by total protein concentration. *P < 0.05. **P < 0.01. Student’s *t-test*.

After stably expressing TOX3 or the control, the cell viability of KGN or SVOG cells was evaluated using the CCK-8 assay. As shown in **Figure 2A** and **Figure S1A**, induced expression of TOX3 in granulosa cells promoted cell viability. Since TOX3 has been reported to be a survival factor for cancer cells in the nervous system (12), it was further examined whether TOX3 protects granulosa cells from apoptosis. As shown in **Figures 2B, C** and **Figures S1B, C**, flow cytometry analysis showed a significant decrease in the apoptotic rate in TOX3-overexpressed cells. Consistently, TUNEL staining showed a similar phenotype in cell apoptosis when TOX3 was overexpressed (**Figures 2D, E** and **Figures S1D, E**). Subsequently, the estradiol synthesis ability of KGN cells and SVOG cells were tested and estradiol was detected from the cell culture supernatant and showed a significant increase after TOX3 overexpression (**Figure 2F** and **Figure S1F**). Simultaneously, we knocked out TOX3 *via* CRISPR/Cas9 in KGN cells. Knock out of TOX3 in KGN cells showed a decreased cell viability (**Figure S2A**). Flow cytometry analysis and TUNEL staining showed an increased level of apoptosis rate after TOX3 was knocked out (**Figures S2B–E**). Significantly, the level of estrogen production also decreased in the LV-KO group (**Figure S2F**).

### Transcriptome Profile and GSEA of KGN After TOX3 Overexpression

**Figure 3:**
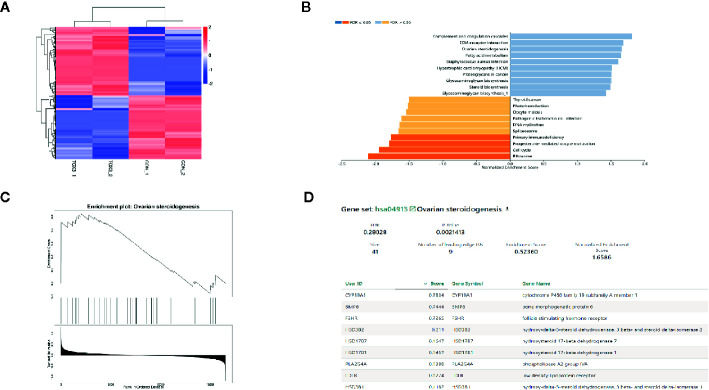
TOX3 regulates steroidogenesis in granulosa cells. **(A)** The total number of DEGs is shown in the heatmap. In total, 1,330 genes were upregulated, shown in red, and 1,178 genes were downregulated, shown in blue. The rows show individual DEGs. Duplicate samples are depicted in columns. Gene expression levels are displayed for each independent sample. **(B)** GSEA showed a significant enrichment or ovarian steroidogenesis. **(C)** The GSEA result of ovarian steroidogenesis. **(D)** The list of gene sets in ovarian steroidogenesis.

RNA was isolated from both LV-TOX3 and LV-CON KGN cells and subjected to sequencing, and 92.51% of the data were mapped to the hg38 reference databases. In total, 2,508 genes were identified as significantly DEGs (|log2FC| >0.3, *P*-value < 0.05), of which 1,330 genes were upregulated and 1,178 genes were downregulated (**Figure 3A**). To investigate the transcriptome changes due to overexpression of TOX3, GSEA was conducted, which summarized and represented specific and well-defined biological processes. This analysis identified ten positively and ten negatively related categories (*P*-value < 0.05). The upregulated gene sets included complement and coagulation cascades, ECM-receptor interaction, ovarian steroidogenesis, and steroid biosynthesis, among others. The downregulated gene sets were associated with ribosomal function, the cell cycle, progesterone-mediated oocyte maturation, and DNA replication (**Figure 3B**). The enriched gene set for ovarian steroidogenesis included several genes that were involved in estrogen synthesis, including *CYP19A1*, *BMP6*, *FSHR*, and *HSD17B1* (**Figures 3C, D**).

### DEG Analysis and Validation

**Figure 4:**
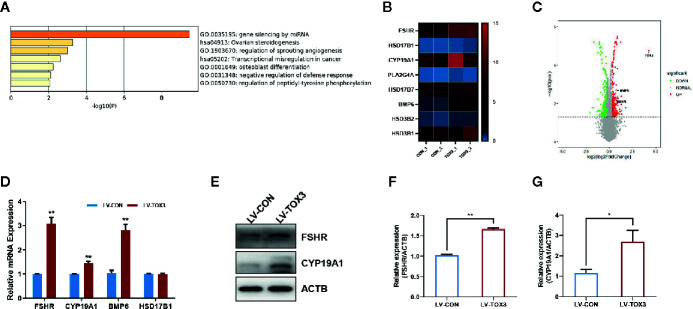
TOX3 transfection leads to increased FSHR, CYP19A1, and BMP6 expression. **(A)** Gene Ontology and Kyoto Encyclopedia of Genes and Genomes (KEGG) analysis performed by Metascape. The ovarian steroidogenesis pathway was significantly enriched after TOX3 overexpression. **(B)** The genes enriched in the steroidogenesis pathways are shown in the heatmap. **(C)** A volcano plot representing DEGs after TOX3 overexpression. **(D)** A qRT-PCR assay was conducted to validate the results of DEGs. The Ct values were normalized to the ACTB levels. **(E)** DEGs were validated *via* western blot. **(F, G)** Protein quantification of FSHR and CYP19A1 was analyzed. Data shown represent three independent experiments and presented as the median ± SEM. *P < 0.05, **P < 0.01. Student’s t-test.

According to the analysis of enriched ontology clusters using the online software Metascape, we identified all significantly enriched processes, including gene silencing by miRNA, ovarian steroidogenesis, regulation of sprouting angiogenesis, transcriptional mis-regulation in cancer, and osteoblast differentiation, among others (**Figure 4A**). To visualize the DEGs that were enriched in the ovarian steroidogenesis pathway, a heatmap was generated, and some genes crucial for estrogen biosynthesis were identified to be upregulated significantly (**Figure 4B**). As shown in the volcano plot, all DEGs involved in estradiol biosynthesis were marked (**Figure 4C**). Upon further validation, we detected a significant upregulation of enzymes responsible for estradiol synthesis, such as *FSHR, CYP19A1*, and* BMP6*, at both the mRNA and protein levels (**Figures 4D–G**). In summary, these results reveal that TOX3 prompts estrogen production *via* fine-tuning of steroidogenesis-related genes in ovarian granulosa cells.

## Discussion

Although TOX3 has been recognized as a susceptible gene in breast cancer for many years, its molecular mechanism in the pathogenesis of breast cancer is still poorly understood. Here, we proposed a possible interpretation. In the present study, overexpression of TOX3 in ovarian granulosa cells, the major cells of endogenous estrogen biosynthesis, was proven not only to promote ovarian granulosa cell proliferation and inhibit cell apoptosis, but also to reinforce estrogen synthesis. Furthermore, we found that TOX3 upregulates expression of CYP19A1, the final and rate-limiting enzyme of estrogen synthesis in granulosa cells (3). These results further support TOX3 in developing of breast cancer.

Estrogen exerts its biological effects through ER activation of estrogen-specific elements, in addition to being able to bind DNA directly (25, 26). Estrogen, therefore, has a wide range of effects on multiple tissues and organs, and it promotes breast cancer development by binding to ERs located in the nucleus of tumor cells (27). For hormone-sensitive breast cancer, blocking both the effects of estrogen on the mammary gland and the production of estrogen is crucial for clinical treatment.

In our work, TOX3 promoted estrogen biosynthesis by upregulating genes related to the development of breast cancer, including *FSHR*, *CYP19A1*, and* BMP6*. Recently, it has been reported that increased expression level of CYP19A1 mRNA in peripheral blood has been shown to be associated with locoregional relapse of breast cancer (28). This indicated CYP19A1 as a potential indicator for the tumor-promoting role of TOX3 in breast cancer. Our study found that the mRNA and protein levels of aromatase were upregulated by TOX3 overexpression in ovarian granulosa cells. In addition to interacting with TOX3, CREB also binds directly to the promoter region of *CYP19A1* and regulates its expression (29). High levels of aromatase expression are associated with breast cancer (30). Therefore, it is worth exploring the role of TOX3 in breast cancer from the perspective of estrogen biosynthesis and function. FSHR is expressed mainly on granulosa cells of the ovary and mediates follicular growth and estrogen synthesis (31, 32). In estradiol biosynthesis, FSHR is indispensable owing to its interaction with follicle-stimulating hormone (FSH), which leads to estrogen metabolism *via* aromatase induction (32). Numerous studies have reported the aberrant expression of FSHR in various tumor cells and peripheral tumor blood vessels (33). It has also been demonstrated that FSHR expression is associated with vascular remodeling in the endothelium of breast cancer (34). Bone morphogenetic proteins (BMPs) are members of the TGF-β superfamily and exert multiple functions during cancer progression (35). In ovaries, BMP6 contributes to dominant follicle selection and estrogen synthesis by modulating FSH actions (36, 37). In this study, we showed that BMP6 was upregulated by TOX3 in KGN cells, which may contribute to the understanding of the roles of TOX3 and BMP6 in the pathogenesis of breast cancer.

Our work in granulosa cells showed that TOX3 exerts an anti-apoptotic effect on ovarian granulosa cells. In previous studies, TOX3 has been demonstrated to be a survival factor (12) or a pro-proliferation factor (14, 38) in several biological processes. TOX3 also upregulates a set of ER target genes that are involved in the cell cycle, cancer progression, and metastasis (14). Currently, few reports are documenting the function of TOX3 in normal breast epithelial cells. However, it is mentioned that TOX3 promoted tumor cell proliferation (13, 39). The expression of TOX3 within the context of breast cancer is reported to be a potential tumor oncogene (14). An association study and subsequent GO/KEGG study found that SNP rs3803662 (TOX3) is an independent prognostic factor for breast cancer, and TOX3 was predicted to be involved in the pathogenesis of breast cancer (40). In addition to breast cancer, TOX3 was also found to be increasingly expressed in tissues of colon cancer and lung adenocarcinoma (41, 42). Therefore, TOX3 may contribute to the process of proliferation in multiple tissues. Consequently, our findings proposed the hypothesis that TOX3 closely correlates with breast cancer development by promoting proliferation and anti-apoptosis of breast tumor cells.

Collectively, this study aimed to explore the effect of *TOX3* on estrogen synthesis from the perspective of estrogen production, in an attempt to link abnormalities in hormone production with the development of breast cancer. After overexpression of TOX3, several crucial key genes and pathways that contribute to estrogen production were upregulated, e.g.*, FSHR*, *CYP19A1*, and *BMP6*. Forced expression of TOX3 also promotes proliferation and inhibits apoptosis of granulosa cells. Nonetheless, there are some limitations in this study. To minimize the limited results due to batch effects of RNA-sequencing, we expanded cutoffs to acquire more differential expressed genes for further analysis and GSEA. Then we screened these genes out with further experimental validation. These findings discuss the possible impact of *TOX3* and steroid hormones on breast cancer from the perspective of extra-mammary tissues such as those in the ovary, thus providing new insights for early prevention and treatment of breast cancer.

## Data Availability Statement

The original contributions presented in the study are publicly available. This data can be found in the Gene Expression Omnibus repository, accession number GSE161341, at https://www.ncbi.nlm.nih.gov/geo/query/acc.cgi?acc=GSE161341.

## Author Contributions

YB and SZ designed the study and revised the manuscript. YM conducted the study and drafted the manuscript. XG and RZ contributed to the interpretation of data. YL verified the data. GL, W-YC, and PL provided the SVOG cell line. All authors contributed to the article and approved the submitted version.

## Funding

This work was supported by the National Key Research and Development Program of China (2018YFC1004303, 2018YFC1003200. 2017YFC1001000, 2017YFC1001504, 2017YFC1001600), Basic Science Center Program of NSFC (31988101), National Natural Science Foundation of China (31871509, 81871168, 31900409, 82071610, 82071606), National Natural Science Foundation of Shandong Province (JQ201816, ZR2016HQ38, ZR2019MH085), Innovative research team of high-level local universities in Shanghai (SSMU-ZLCX20180401).

## Conflict of Interest

The authors declare that the research was conducted in the absence of any commercial or financial relationships that could be construed as a potential conflict of interest.
