# Supplementary figures and images for: TOX3 Promotes Ovarian Estrogen Synthesis: An RNA-Sequencing and Network Study
Source: Front Endocrinol (Lausanne). 2021 Feb 24;11:615846. doi: 10.3389/fendo.2020.615846 (PMC7945945; doi:10.3389/fendo.2020.615846)

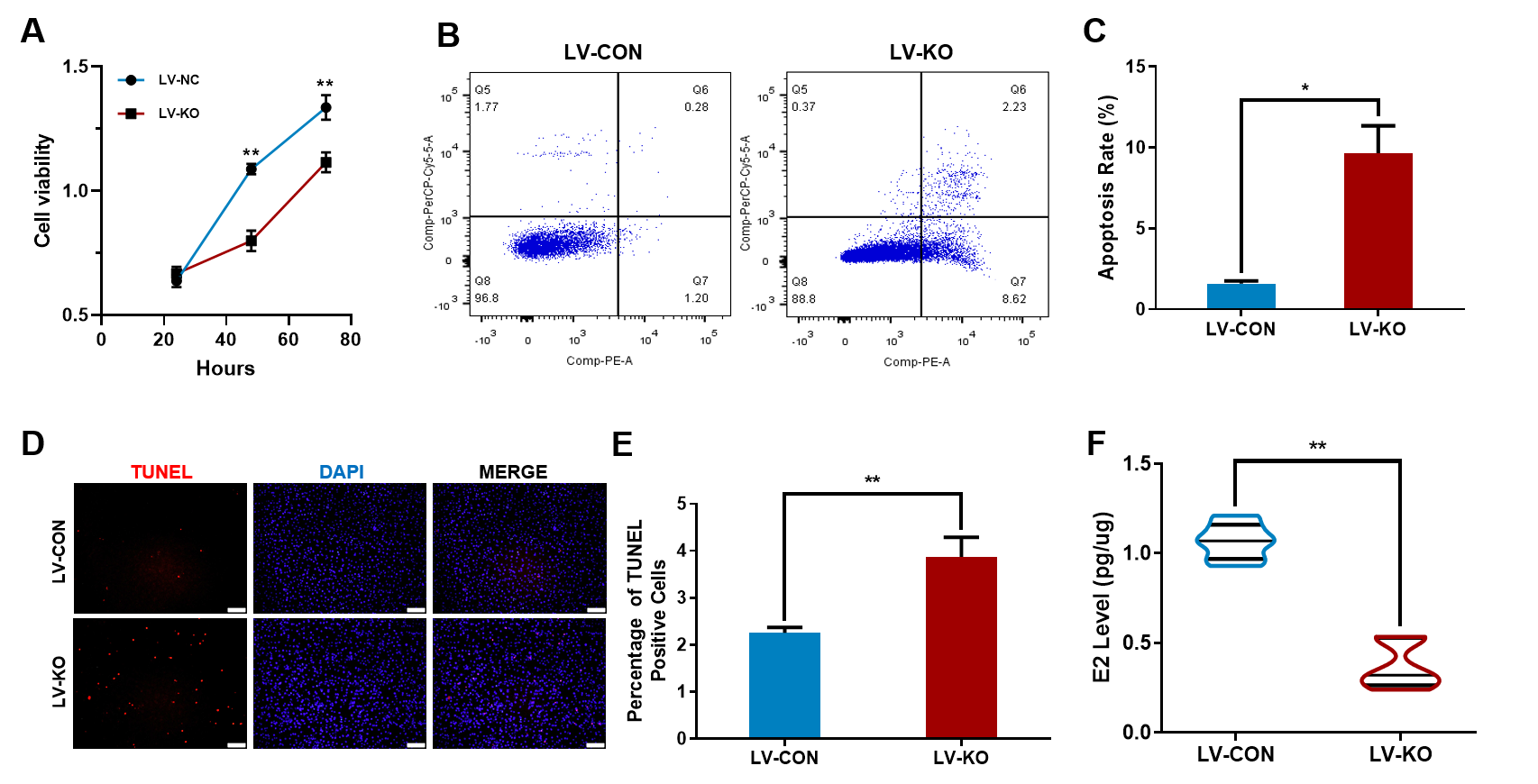

Supplement: Supplementary file 4 [file Image_1.tif]

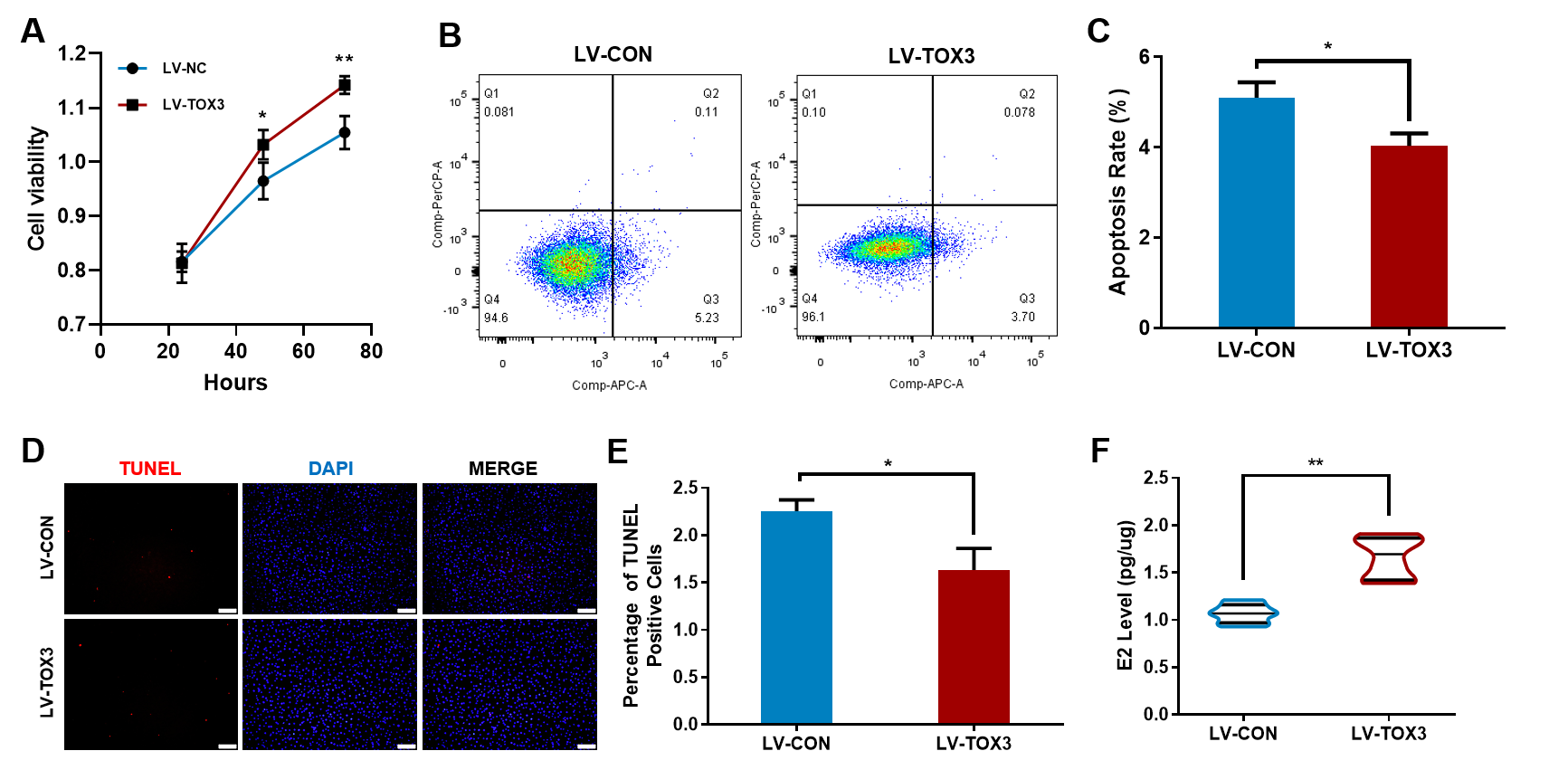

Supplement: Supplementary file 5 [file Image_2.tif]
